# Brown Adipose Crosstalk in Tissue Plasticity and Human Metabolism

**DOI:** 10.1210/endrev/bnz007

**Published:** 2019-10-15

**Authors:** Camilla Scheele, Christian Wolfrum

**Affiliations:** 1 Novo Nordisk Foundation Center for Basic Metabolic Research, Faculty of Health and Medical Sciences, University of Copenhagen, Denmark; 2 The Centre of Inflammation and Metabolism and Centre for Physical Activity Research Rigshospitalet, University Hospital of Copenhagen, Denmark; 3 Institute of Food, Nutrition, and Health, ETH Zürich, Schorenstrasse, Schwerzenbach, Switzerland

## Abstract

Infants rely on brown adipose tissue (BAT) as a primary source of thermogenesis. In some adult humans, residuals of brown adipose tissue are adjacent to the central nervous system and acute activation increases metabolic rate. Brown adipose tissue (BAT) recruitment occurs during cold acclimation and includes secretion of factors, known as batokines, which target several different cell types within BAT, and promote adipogenesis, angiogenesis, immune cell interactions, and neurite outgrowth. All these processes seem to act in concert to promote an adapted BAT. Recent studies have also provided exciting data on whole body metabolic regulation with a broad spectrum of mechanisms involving BAT crosstalk with liver, skeletal muscle, and gut as well as the central nervous system. These widespread interactions might reflect the property of BAT of switching between an active thermogenic state where energy is highly consumed and drained from the circulation, and the passive thermoneutral state, where energy consumption is turned off. (Endocrine Reviews 41: XXX – XXX, 2020)

## Graphical Abstract

**Graphical Abstract:**
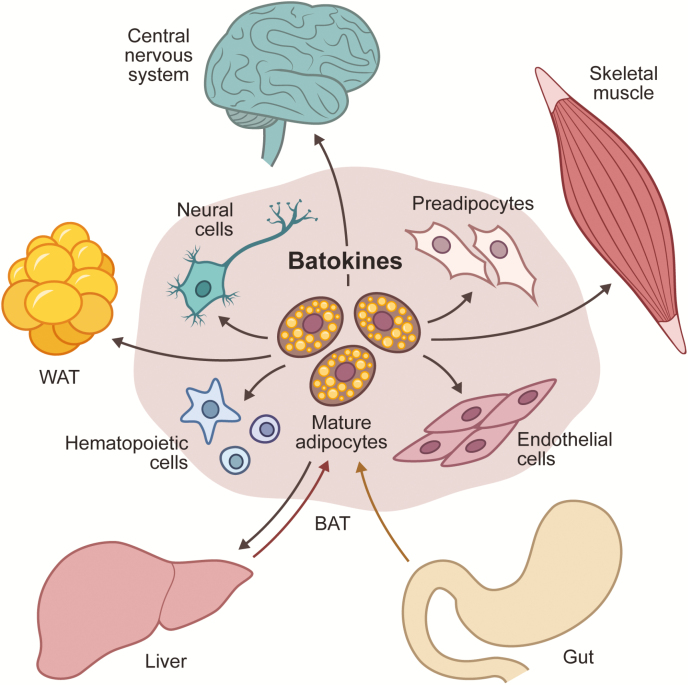

**ESSENTIAL POINTS**

- Recruitment of brown adipose tissue (BAT) includes secretion of factors, known as batokines
- Batokines act on cells within the adipose tissue and promote adipogenesis, angiogenesis, immune cell interactions, and neurite outgrowth
- BAT is part of an interorgan crosstalk with liver, skeletal muscle, and gut as well as the central nervous system

It is well established that adipose tissue is an important contributor to the regulation of energy metabolism through organ crosstalk. This is exemplified by adipokines secreted from mainly white adipose tissue (WAT) including leptin and adiponectin, which are released as a response to a certain energetic state (1, 2). Since the discovery that adult humans have functionally competent brown adipose tissue (BAT) (3–7), multiple studies have addressed its function in energy metabolism. It was shown that in adult humans active BAT content is negatively correlated with body mass index (BMI) (4, 5) and in vivo studies suggest a functional role of BAT in whole-body metabolism (8–12). Importantly, studies using fluorine-18 fludeoxyglucose (^18^F-FDG)-positron emission tomography/ computed tomography (PET/CT) scanning following cooling suggest that not all individuals have cold-responsive BAT, revealing a loss of a potentially important metabolic function in a large part of the population (4, 13–16). Although minor increases in BAT response following repeated cold exposure have been reported (11, 17, 18), BAT activation is clearly associated with age (15, 16) and no satisfactory approach to efficiently restore functional BAT in aging and obese subjects has been reported to date. Studying secreted factors from metabolically active BAT, known as batokines, offers an alternative approach to identify novel drug targets for metabolic regulation. Batokines have autocrine, paracrine, or endocrine activities, and excellent and extensive reviews on these secreted factors from BAT have recently been provided (19, 20). However, current reports on batokines are mostly based on studies in mouse models, while data on human batokines are limited. Given the obvious differences between mice and humans in terms of thermogenic needs and metabolism, we cannot assume a complete overlap. In this review, we aim to leverage current understanding of the functional spectra of batokines by acknowledging the cellular and energetic complexity of BAT. We provide a structure for the processes and cell types involved in BAT recruitment and we discuss recent findings of BAT crosstalk in whole-body metabolism. Finally, we discuss the plasticity of BAT in the perspective of human physiology and highlight some reported differences in batokines between human and mice.

## Brown Adipose Tissue in Infants versus Adult Humans

Autopsy morphological mapping (21, 22) and post-mortem magnetic resonance imaging (MRI) scanning (23) have revealed substantial amounts of BAT in human infants, suggesting that this fat is a necessary regulator of body temperature in early life when muscle shivering is not yet developed (24). This heat production is probably also the most important function in small mammals, such as the widely studied rodents (25). An extensive post-mortem mapping of brown adipocytes in human adipose tissues from infancy and up to 80 years of age revealed a gradual change in BAT morphology (22). During the first decade of age, multilocular adipocytes were abundant and contained only small amounts of lipids. Although declining in frequency among individuals, this phenotype was found up to the age of 20. After this, multilocular adipocytes were still found; however, these cells were more lipid filled (22).

In adult humans, the superficial interscapular BAT has disappeared. However, active BAT can, especially in younger adults, be found in the superficial supraclavicular depot as well as the deeper depots, aligning the kidney and the spinal cord up to the neck (3–7). A direct quantitative comparison between infants and adults is difficult to perform due to the limitations in the current methodology of determining amounts of active BAT, which relies on acute cooling in combination with injection of ^18^F-FDG, a radioactive glucose tracer, prior to PET and CT scanning. While this approach can be used in adults, it cannot be performed in infants. The gradual changes in BAT appearance adds to the complexity in direct comparisons of BAT abundance between infants and adults.

Interestingly, similarly to small mammals (25), adult BAT activity is increased in association with a meal and is linked to uptake of circulatory fatty acids and glucose (26), suggesting a cold-independent, albeit still sympathetically regulated, role of BAT in promoting metabolic homeostasis. Notably, as mentioned above, the morphology of BAT in adult humans differs from that of infants and is characterized by a heterogeneous mixture of unilocular and multilocular adipocytes with a higher fat content than is seen in the homogeneously multilocular infant BAT (6, 22, 27, 28). In this respect, infant BAT resembles intrascapular BAT of mice housed at temperatures below thermoneutrality, while BAT from adults has the morphology of inguinal WAT of mice housed under cold conditions or interscapular BAT of mice housed at thermoneutrality (29). Thus, BAT morphology seems to reflect the usage or activity of the tissue. It is a long-standing debate whether interconversion occurs between brown adipocytes and white adipocytes (30) and whether this constitutes an uni- or a bidirectional process. It was shown that differentiation of thermogenic adipocytes in WAT during cold acclimation occurred through de novo recruitment of preadipocytes (31). However, it remains unclear whether white interconversion of brown adipocytes is due to a cell identity switch or due to the formation of an “inactive” “or dormant” brown adipocyte state (32). However, a recent study on perirenal BAT in adults demonstrates the presence of unilocular adipocytes which are mitochondrial brown fat uncoupling protein 1 (UCP1) positive, and the stromal vascular fraction of adipose biopsies in this region contained preadipocytes that differentiated into thermogenic adipocytes in vitro (27). Altogether, these findings emphasize the plasticity of BAT and seem to reflect a continuous environment-dependent adaptation of this tissue. While sympathetic activity probably is a major overarching regulator of this plasticity, downstream mediators, ie, “batokines,” acting specifically on the different cell types composing BAT, could be valuable tools for increasing the amount of mature active brown adipocytes in adult humans.

### BAT plasticity and batokines

**Figure 1.**
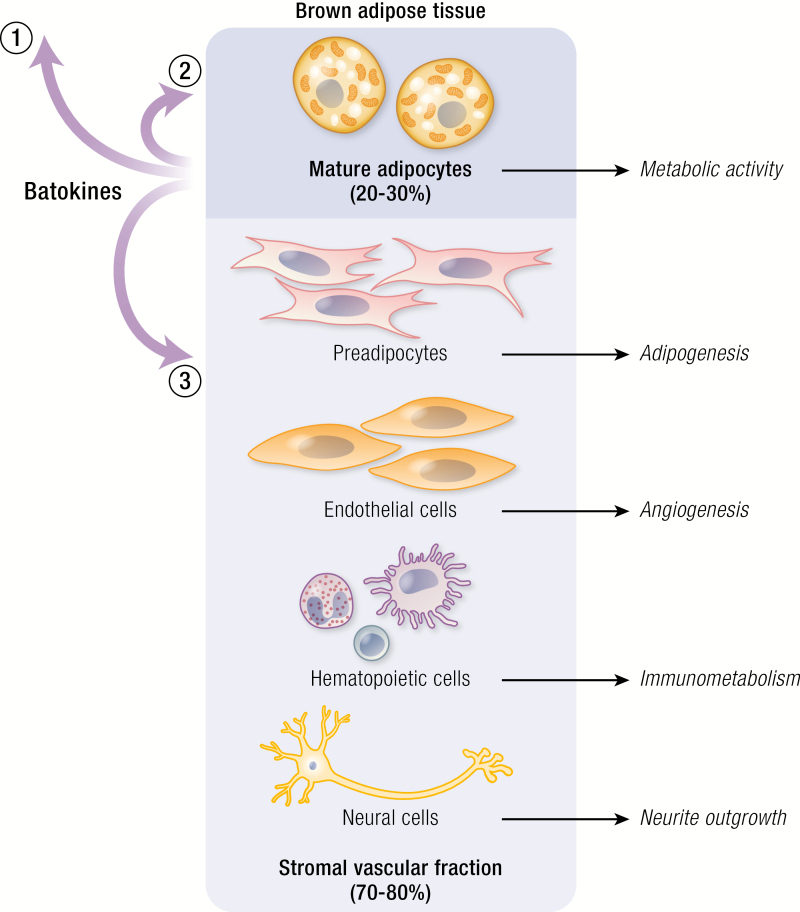
Batokine-mediated processes during brown adipose tissue (BAT) recruitment. Adipose tissue is composed of mature adipocytes (20% to 30%) and a stromal vascular fraction with a dynamic composition of preadipocytes, endothelial cells, hematopoietic cells and neural cells. Batokines are produced by the mature brown adipocytes and can (1) act on other tissues in an endocrine fashion; (2) enhance thermogenic activity; (3) induce processes involved in BAT recruitment including adipogenesis, angiogenesis, immunometabolism, neurite outgrowth.

BAT is composed of 20% to 30% mature adipocytes (33) and a stromal vascular fraction in which adipose stem cells, preadipocytes, endothelial cells, hematopoietic cells, and neural cells reside (Fig. 1). In WAT, which is similarly composed (33), it has been observed that major differences in adipose-resident immune cell population occurs in relation to obesity, providing systemic low-grade inflammation (34, 35). Less is known about changes to cellular composition in BAT during obesity. However, studies in adult humans show that BAT interacts with the environment and adapts to thermal conditions (11, 17, 18, 36, 37). Cold acclimation requires multiple cell types within the adipose tissue including the following. (1) Metabolic activity of the mature adipocytes, which is an acute response mediated by adrenergic signaling, resulting in lipolysis of intracellular triglycerides fueling an increased mitochondrial uncoupled respiration (25). (2) angiogenesis, which allows an increased influx of oxygen for the uncoupled respiration and glucose and lipids to replenish energy sources. (3) Immunometabolism, a complex interplay between immune cells and adipocytes and, finally, (4) neurite outgrowth enhancing upcoming sympathetic activation and afferent signaling to the central nervous system (CNS). Several cell types thus coexist within BAT that are all affected by and contribute to cold acclimation. Multiple growth factors, cytokines, and chemokines have been shown to be released from both mature and thermogenically activated brown adipocytes (19, 20, 38, 39). Determining which cells these factors are targeting and how they affect the cells could provide us with keys to leverage our understanding about how childhood levels of BAT can be restored in adult humans.

### Metabolic activity in thermogenic BAT

Batokines proposed to increase BAT activity are frequently linked to enhancing energy uptake of glucose and lipids (19, 40). Some batokines have been shown to be released from activated brown adipocytes and to act in a feedback loop to promote BAT activity. For example adenosine, a biproduct of norepinephrine production, is released locally by BAT and has been shown to activate BAT in rodents (41). The physiological effect of adenosine was further explored by intravenous injections of adenosine in humans, and subsequent estimation of BAT activity by measuring perfusion using PET/CT imaging with [^15^O]H_2_O. Strikingly, the effect of adenosine was higher than the effect of cold, highlighting the importance of adenosine in BAT activation (42).

Early studies identifying active BAT in adult humans could declare an increased uptake of the glucose tracer, ^18^F-FDG, from the circulation following acute cooling. More recent studies have applied so-called dynamic PET/CT scanning, where the rates of glucose uptake can be determined, rather than just a snapshot of glucose uptake at a given timepoint (43, 44). This allows more physiologically relevant comparisons between different subject groups. Using this approach, the response in BAT glucose uptake rate to acute cold challenge could be determined to be between 80 ± 14 nmol g^−1^ min^−1^ (in noncold-acclimated subjects) and 209 ± 50 nmol g^−1^ min^−1^ (in cold-acclimated subjects) (45).

Glucose is however not considered to be the main substrate for BAT thermogenesis in adult humans. Interestingly, a study using microdialysis of supraclavicular BAT demonstrated that a large portion of the glucose absorbed during cooling is subject to anaerobic metabolism and released as lactate (46). Cold-induced sympathetic activity results in increased levels of circulating fatty acids from white adipose tissue (47–50) and studies using the PET tracer 14(R,S)-[^18^F]-fluoro-6-thia-heptadecanoic acid (^18^FTHA) (a long-chain fatty acid analog) demonstrates a robust uptake. Although the uptake of non-esterified fatty acids (NEFA) in BAT during cold activation was associated with BAT thermogenesis (26), this tracer is trapped in the mitochondrial matrix and thus not suitable as a marker for estimating the extent of oxidative metabolism. However, it can be used for estimation of relative fatty acid uptake between tissues. In this respect, it was determined that during cooling, BAT dietary fatty acid uptake was 2-fold higher than in the neck subcutaneous WAT, and 3-fold higher in skeletal muscle (51).

Nevertheless, it has been suggested that BAT of adult humans utilize intracellular sources of energy as a first choice, for inhibition of lipolysis resulted in increased shivering, ie, a switch to muscular thermogenesis (48).

In sharp contrast, rodents seem to be more dependent on energy uptake from the circulation for providing enough fuel for BAT thermogenesis. The energy uptake of active BAT is high and results in a substantial effect on triglyceride clearance (52). Furthermore, a knockout model of adipose triglyceride lipase, a key enzyme in the lipolysis of intracellular fat storage, demonstrated an unchanged nonshivering thermogenesis. These data suggest that energy uptake from the circulation provides sufficient fuel for BAT activity in rodents (53).

**Figure 2.**
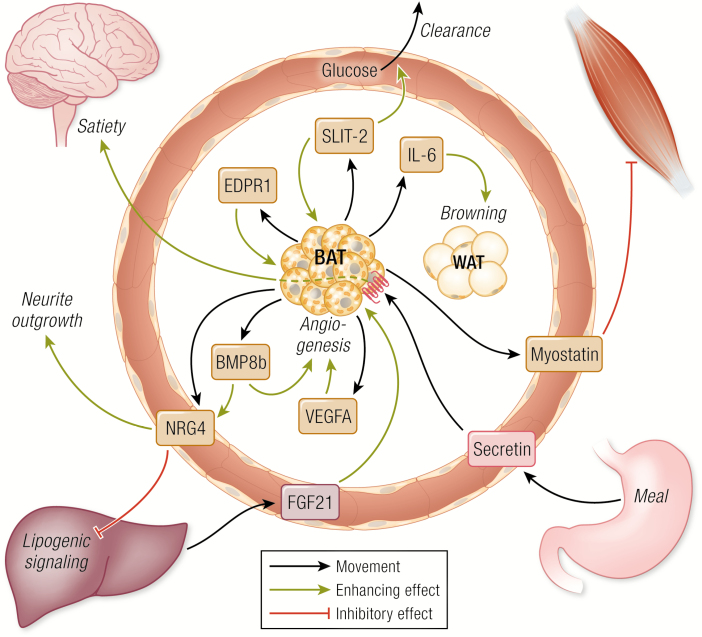
Brown adipose tissue (BAT) crosstalk in metabolism. Batokines and other factors promoting crosstalk that could be relevant for human metabolism. A detailed description of all factors is provided in the text.

This discrepancy between human and rodents could be related to substantial differences in intracellular lipid storage where adult humans have much more stored triglycerides than the standard mouse model. Interestingly, housing mice at thermoneutrality on a high-fat diet, provides a “humanized BAT mouse” with comparable lipid accumulation in BAT (54) and thus providing a more suitable model for studying BAT substrate metabolism. It is possible that thermogenic substrate uptake is supported by factors secreted from brown adipocytes, “BAT-activity batokines,” which might act directly to enhance energy uptake, allowing increased thermogenic activity or de novo lipogenesis. Slit homolog 2 protein (Slit2) is an example of such a batokine (Fig. 2) (40). It was found that Slit2 is produced, cleaved into Slit2-C, and secreted from murine beige adipocytes in response to overexpression of Prdm16, a cotranscription factor of the thermogenic gene expression program (55). When added as a recombinant peptide, Slit2-C amplified energy expenditure and glucose clearance. In support, a later study demonstrated that at least the precursor protein, SLIT2, was detected in human plasma and negatively correlated with plasma glucose levels, raising the idea of a possible role in glucose metabolism in humans, although further studies will be required to confirm this idea (Fig. 2) (56). Slit3 and Epdr1 were also shortlisted among the Prdm16- induced proteins in (40) and might belong to the same group of batokines. EPDR1 (Fig. 2) and SLIT3 were later identified in a study of the human brown adipocyte secretome (38), and EPRD1 was found to be important for differentiation of human thermogenic adipocytes (38). Interestingly, the crystal structure of EPDR1 was recently published, revealing 2 putative lipid-binding domains (57). A full understanding of the function of these proteins in human metabolism remains to be established.

### BAT adipogenesis and the TGF-β signaling pathway

Preadipocytes originate from mesenchymal stem cells and both populations are present in the adipose tissue. Upon cold-induced BAT recruitment, norepinephrine stimulates proliferation of brown preadipocytes, and apoptosis of brown adipocytes is attenuated, resulting in an accumulation of active brown adipocytes as part of a cold acclimation (58). Preadipocytes derived from the stromal vascular fraction of adipose tissue differentiate in vitro in response to the same differentiation cocktails regardless of whether they originate from WAT or BAT (59). However, preadipocytes originating from BAT are preprogrammed to differentiate into functional brown adipocytes, whereas preadipocytes derived from WAT differentiate into white adipocytes (28). This seem to occur through a sequential interaction between endogenous transcription factors and autocrine growth factors (60). Interestingly, not only norepinephrine but also batokines secreted from mature adipocytes activated by norepinephrine might influence the programming.

**Figure 3.**
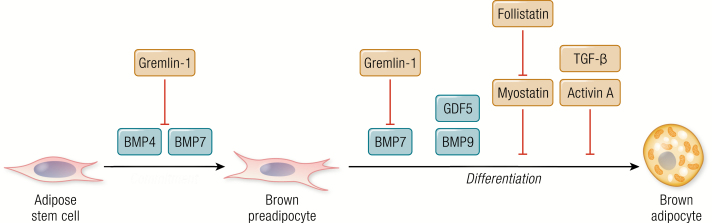
Transforming growth factor (TGF)-β-associated batokines in brown adipogenic commitment and differentiation. Factors part of the TGF-β signaling and negative regulators are shown.

Many secreted factors affecting brown adipogenesis have been reported (19, 38), while proteins involved in the transforming growth factor (TGF)-β signaling pathway are strikingly dominating. This signaling pathway is involved from early adipogenic commitment and throughout the differentiation into mature adipocytes (Fig. 3) (19, 33, 61). A subgroup in the TGF-β superfamily are the bone morphogenic proteins (BMPs). These developmental growth factors have been described to be important for regulating brown adipocyte commitment of preadipocytes or mature adipocytes in both brown and white adipose tissue (62, 63). BMP4 is one of the most studied members of the family in terms of adipogenic differentiation and is important for both white and brown adipogenic commitment (64, 65). Together with BMP7, BMP4 promotes early commitment towards the brown adipose lineage (66). However, whereas BMP7 also facilitates later phases of brown adipogenesis (66), the literature on the role of BMP4 as selective for brown or white adipogenesis is conflicting (66–68); the different studies are outlined and discussed in (69). The contradictory reports likely reflect the temporal complexity of brown and white adipogenesis. BMP4 and BMP7 share the inhibitor Gremlin-1, which is secreted from preadipocytes (68). Another subgroup of the TGFβ superfamily are the growth differentiation factors (GDFs). Among these, GDF5 and GDF2 (also known as BMP9) have been reported to enhance brown adipogenesis (70), whereas GDF8 (also known as myostatin) has an inhibiting effect (71). This effect is likely related to interactions with the brown adipogenesis stimulator, follistatin (72), as myostatin and follistatin are antagonizing factors (73). Finally, TGF-β and Activin A were shown to inhibit brown adipogenesis by blocking the induction of SREBP-1, an inducer of adipogenesis (74) (Fig. 3).

### Angiogenesis and BAT recruitment

Although BAT relies on innervation for sympathetic activation, it cannot be excluded that hormonal adrenergic stimulation also plays an important role. In humans, this is clearly exemplified by studies of patients with pheochromocytoma, a catecholamine-producing tumor in the adrenal gland. The high circulating endogenous levels of norepinephrine in association with this disease results in extensive BAT recruitment (30, 75). Notably, the more vascularized visceral adipose depots also acquired thermogenic morphology (30) and glucose tracer ^18^F-FDG uptake (75), while no browning was observed in the subcutaneous adipose depots. Further evidence of vascularization as a key mechanism in BAT activity was the finding that depletion of VEGFA in murine BAT resulted in whitening (76), while overexpression of VEGFA induced browning in WAT (77). A similar capillary rarefaction resulting in whitening was observed in BAT of diet-induced obese mice. Although a similar rarefaction in BAT of obese humans has not yet been reported, it might contribute to the reduced glucose tracer uptake observed in obesity (4, 5).

As mentioned above, initial reports described a negative association between BAT activity and BMI (4, 5), pointing to a functional inactivation of BAT in obesity. It was later determined that ^18^F-FDG-positive BAT volume is smaller in individuals with obesity and type 2 diabetes (47). Furthermore, by applying dynamic PET/CT scanning, BAT glucose uptake rates during acute cold exposure were estimated to 90–120 nmol g^−1^ min^−1^ in healthy subjects and 35 nmol g^−1^ min^−1^ in obese individuals (45). Thus, if only using glucose uptake as an estimate of BAT activity, these data would support the idea that BAT activity is reduced in obesity.

However, other observations suggest that the reduction in glucose uptake is not reflecting impaired BAT lipid metabolism or BAT thermogenesis. This was clearly shown in a human study of older overweight individuals with and without type 2 diabetes compared with young healthy subjects. In this study, no reduction in BAT NEFA uptake and thermogenic capacity was observed on acute cold exposure in the older subjects, although glucose uptake, as expected, was found to be substantially reduced (47). Interestingly, reduced BAT glucose uptake is associated with increased BAT fat content (44, 47).

Moreover, whereas human BAT is responsive to both insulin and cold (78), this response is severely blunted in obesity (79). Interestingly, BAT activation associated with cerebral activity in lean subjects, but was blunted in obese subjects (80). Taken together, studies in humans suggest an impairment of BAT activation capacity during obesity which could be due to regulation both at a central and peripheral level.

Indeed, based on the described observations in rodents, vascular rarefaction might be part of the reduced BAT activity in humans. Importantly, human brown adipocytes produce and secrete both Vascular endothelial growth factor A (VEGFA) and Vascular endothelial growth factor D (VEGFD), although VEGFD has not yet been explored in gain- or loss-of-function studies (38). Importantly, proangiogenic factors also stimulate the proliferation and development of human thermogenic preadipocytes (81), further emphasizing angiogenesis as a central mechanism in BAT recruitment.

Another interesting factor produced by mature brown adipocytes and belonging to the TGFβ-superfamily, is BMP8b (82). BMP8b was shown to promote ancillary networks for BAT recruitment by coordinately stimulating both vascular and nervous networks (83). The production of Neuregulin-4 (NRG4), a batokine-stimulating neurite outgrowth, was enhanced by BMP8b as were also angiogenic factors, including VEGFA (83). NRG4 has also been shown to stimulate angiogenesis in adipose tissue, further underlining the synchronization of vascularization and neurite outgrowth within BAT (83).

### Neurite outgrowth in BAT

BAT contains neuronal cells and innervation is a key mechanism in BAT recruitment. Increased nerve endings will increase exposure to norepinephrine during acute activation of BAT. This mechanism was recently confirmed in BAT of adult humans using ^11^C-meta-hydroxyephedrin and ^15^O-water PET imaging (84). It was found that the level of sympathetic innervation was clearly associated with levels of functional BAT (84). This was also closely connected with vascularity, as blood flow was the best predictor of both sympathetic innervation and functional BAT (84). As mentioned above, the recruitment and remodeling of a neurovascular network in BAT is coordinated through induction of BMP8b, which is secreted from the activated mature brown adipocyte (82). BMP8b induces the production of secreted NRG4, which promotes sympathetic axon growth and branching in vitro and at the same time induces angiogenesis as outlined above (83). In adult humans, neuronal branching and communication with the central nervous system in the context of BAT function remains to be studied. Besides modulation of local neural networks, the possibility exists that BAT also secrets hormones that act on the central nervous system like the WAT-produced satiety hormone, leptin. Currently, however, there is no clear evidence for such a BAT-specific hormone. Alternatively, BAT, with its extensive innervation, communicates directly through afferent neural networks. This idea is supported by a novel study discussed below.

### BAT and immunometabolism

Since the finding in 2003 that immune cells reside in WAT (34, 35), the interactions between adipocytes and immune cells has been widely studied, and inflammation, fibrosis, and impaired adipogenesis have been described as key players in adipose tissue dysfunction (85). Studies of dysfunctional WAT in obesity (86) and insulin resistance (87) reveal a reduction in sensitivity to β-adrenergic stimulation and subsequent lipolysis, leading to a progressive lipid accumulation. Similar dysregulations might occur in BAT, which might explain the observed decreased BAT activity with obesity (4, 5). During recent years, the role of BAT resident immune cells in regulating BAT activity and recruitment has been intensively studied and discussed. CXCL14 has been highlighted as a novel batokine secreted by thermogenically activated brown adipocytes, which recruits alternatively activated macrophages into BAT. This was in turn shown to promote adaptive thermogenesis and result in M2-mediated browning of the murine inguinal depot. This finding relates to an intense academic debate on the role of macrophages on BAT and beige fat recruitment and thermogenic activity (88). As pointed out above, in pheochromocytoma patients excess levels of norepinephrine result in BAT recruitment and “browning” of visceral fat. Norepinephrine is produced in the locus coeruleus in the brain as a neurotransmitter and can also be synthesized by the prolongation of the sympathetic nervous system, the adrenal gland, from which it is released as a hormone. A recent discussion has centered around the capacity of alternatively activated macrophages for producing norepinephrine, providing a direct link between immune cells and thermogenesis and BAT recruitment (89, 90). Evidence strongly arguing against this mechanism was presented in a follow-up study (91). In this context, a separate mechanism was presented where sympathetically associated macrophages contribute to the clearance within BAT, by importing and metabolizing norepinephrine (92). Others in the meantime reported that macrophages promote BAT innervation (93). With focus on the secretome of BAT it was found that human brown adipocytes had a differentially regulated complement system compared with human white adipocytes (38), a finding replicated in murine adipocytes with a similar approach (39). These findings raise the idea that BAT has other immunological properties than WAT which could be associated with the presence of BAT at locations in adult humans not evidently requiring thermogenesis. This includes, eg, the visceral fat regions surrounding the kidney (14, 94) and heart (95). Altogether, these studies indicate close interactions between immune cells and the other cells present in BAT.

## BAT Crosstalk With Other Organs

### BAT-mediated satiety signal from the gut to the central nervous system

A crosstalk between BAT, WAT, and the central nervous system (CNS) has been described to occur via a circuit of sensory neurons in WAT and sympathetic innervation of BAT (96). BAT also exhibits afferent neurons, which feedback on brain regions controlling sympathetic activation of BAT (97). The CNS–BAT circuit is organized in a functional network affected by fluctuations in temperature and energy homeostatis, and is in turn adjusting metabolic regulation through sympathetic nerve activity of BAT (98). Interestingly, a recent study discovered that secretin, a gut peptide, was transported to BAT via the circulation and could induce prandial thermogenesis and increased glucose uptake in BAT, in turn mediating a satiety signal to the CNS via afferent nerve fibers (99). This raises the conceptual idea of BAT as an enhancer of satiety/hunger signals from the gut and proposes afferent signaling as a possible additional important function of BAT. Indeed, postprandial induction of thermogenesis has been recorded in adult humans at a similar intensity as when stimulated with mild cold (26). It is well established that food intake increased at the onset of the active phase in rodents (100), coinciding with increased BAT activity (101). These everyday fluctuations in BAT activity should require strict regulation. Whereas fatty acid availability and release and uptake of norepinephrine certainly are major regulators of BAT activity (25), it is likely that multiple mechanisms and factors are involved in controlling this energy-consuming process.

### Batokine-mediated prevention of hepatic lipogenesis

Risk factors for excessive storage of fat in the liver, besides alcohol overconsumption, include obesity and diabetes. This can lead to nonalcoholic fatty liver disease (NAFLD), which in turn is associated with severe complications including cirrhosis, cardiovascular disease, and hepatic carcinoma (102). BAT activity will increase fatty acid oxidation and plasma clearance, but could there be a direct crosstalk between brown adipocytes and hepatocytes? Interestingly, NRG4, mediating both neurite outgrowth and angiogenesis as described above, has been reported to increase during brown adipocyte differentiation while decreasing in adipose tissue during human obesity (103). It was found that NRG4 attenuated diet-induced insulin resistance and a specific effect was reported in the liver. Here, NRG4 prevented de novo lipogenesis through induction of ErbB3 and ErbB4 signaling in hepatocytes (103). In line with these findings, decreased serum levels associated with NAFLD in children with obesity (103). Importantly, how much of the NRG4-dependent antisteatosis effect that is mediated by inhibition of de novo lipogenesis and how much that is mediated by increased fatty acid oxidation by BAT is currently not known.

### The liver-derived hormone FGF21 increases BAT thermogenesis

FGF21 is another factor connecting liver with BAT. This hormone is produced and secreted by the liver and mediates a range of metabolic effects on whole-body physiology (104). In rodents, Fgf21 is secreted from BAT in response to thermogenic activation (105). In human brown adipocytes, the expression of FGF21 is close to undetectable, and FGF21 was not detected in the secretome of human brown adipocytes (38). This discrepancy might be related to the differences between interscapular BAT (usually analyzed in mice) and supraclavicular BAT (usually analyzed in human adults) (23) and could in turn be related to the well-established differences in the limited recruited BAT in adult humans compared with mice housed at room temperature, a temperature below murine thermoneutrality (106). Nevertheless, FGF21 has been shown to enhance thermogenesis in human brown adipocytes derived from the neck (107), and as circulating levels of FGF21 are elevated in response to ingested glucose (108) and alcohol (109) but not cold (107) this protein is likely to be involved in liver–BAT crosstalk in humans; however, whether is acts as a cold-regulated factor remains to be studied. Recent reports have investigated the causal link between FGF21-mediated browning and the loss of body weight in mice (110–112). Interestingly an examination in UCP1 knockout mice (UCP1 KO) suggests that the effects of FGF21 on weight loss and glycemic control (111, 112) as well as on thermogenesis (110) are UCP1 independent. The role of FGF21 in energy expenditure has been discussed in depth (113). It should be noted that food intake in response to FGF21 is reduced in UCP1 KO and the lack of weight loss is thus intriguing (113). In conclusion, more detailed studies will be required to delineate both the contribution of BAT to circulating FGF21 levels and the role of BAT in mediating the anti-obesogenic effects of FGF21.

### Loss of IRF4 and myostatin secretion from BAT attenuates muscle capacity

Following infancy, skeletal muscle takes over the role as the major site of thermogenesis from brown adipose tissue in humans (21, 24). Myocytes and brown adipocytes are both derived from mesenchymal stem cells and share similarities in precursor transcriptomes (114), in terms of being mitochondria-rich and insulin-sensitive cells with mitochondria-dependent activity levels that can be switched on and off (ie, thermogenesis in BAT and contraction in muscle). Despite these similarities, there is not yet any documentation of a crosstalk between BAT and skeletal muscle in humans. In rodents, however, a recent study demonstrated BAT to muscle crosstalk, where a loss of the transcription factor IRF4 in BAT results in increased transcription of myogenic genes, including myostatin (115). In skeletal muscle, this alteration in BAT resulted in reduced mitochondrial function and diminished exercise capacity. The upregulation of myogenic genes in BAT has previously been reported in response to activation of the AgRP neurons which impaired insulin-stimulated glucose uptake in BAT, while inhibition of myostatin increased insulin sensitivity (116). Thus, BAT upregulation of myostatin affects BAT insulin sensitivity as well as muscle status and might serve as a mechanism in central regulation of glucose homeostasis. To date, it remains elusive if a similar mechanism exist in humans. From a larger perspective, these findings suggest that alterations in BAT affect muscle, and it opens the possibility for changes in skeletal muscle that could also influence BAT.

### Interleukin-6-mediating browning of WAT?

Interleukin (IL)-6 is a multifunctional cytokine with contextual functions either as part of an acute inflammatory response or as a myokine promoting muscle glucose uptake and myogenesis (117, 118). IL-6 has also been proposed to play a role in exercise-induced “browning” of WAT as expression of marker genes for this process was blunted in IL-6 knockout mice (119). Browning represents the mechanism where WAT acquires a thermogenic phenotype (120, 121). In further support of IL-6 mediating browning, the beneficial effects on glucose homeostasis of BAT transplantation into the WAT depot disappeared when transplanting BAT from IL-6 knockout mice (122). The precise mechanism for IL-6-mediated browning and whether it is relevant in humans remains to be investigated. IL-6 is upregulated in the circulation following acute exercise (123), and multiple studies have assessed the effect of exercise on browning in humans. Interestingly multiple studies point to the fact that browning is not induced by exercise in human subcutaneous fat (124–127). A reason for this might be the limited innervation and vascularization of the subcutaneous abdominal adipose depot. Given the browning capacity of the visceral adipose depot observed in patients with pheochromocytoma, it could be argued that this depot would be the most relevant to study when addressing the occurrence of WAT browning in adult humans. However, obtaining human visceral adipose tissue is not trivial and is mostly done in conjunction with a surgical intervention.

## Perspectives

**Figure 4.**
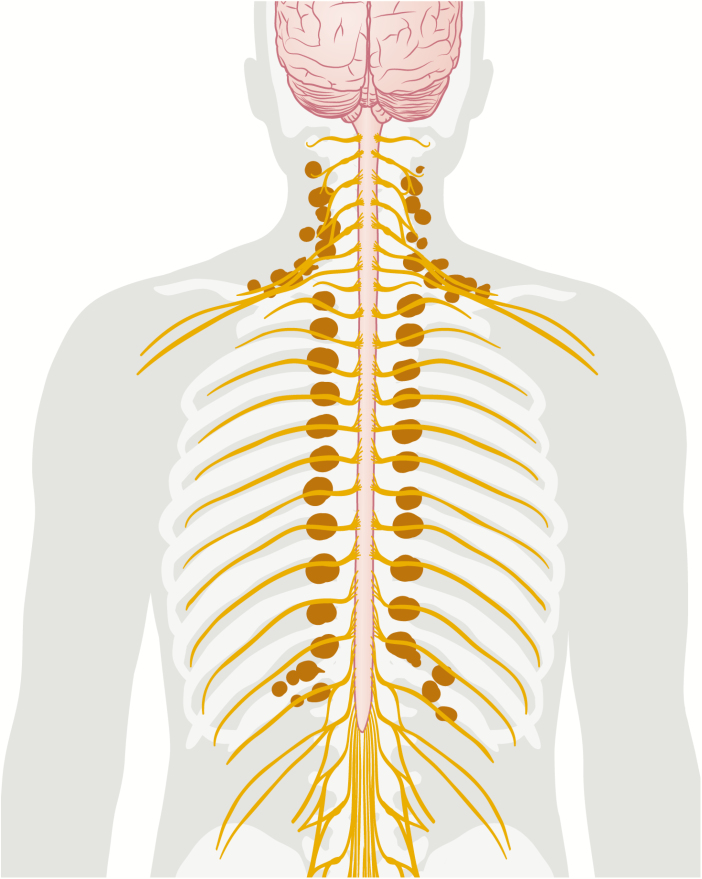
A cartoon showing representative brown adipose tissue (BAT) occurrence in relation to the central nervous system in an adult human.

Adult humans have BAT and this tissue plays a role in whole-body metabolism (16); recent studies strongly suggest a whole-body crosstalk which involves BAT. Given the metabolic properties of WAT, it is intriguing to assess whether BAT secretes an equally potent hormone as the key metabolic regulator leptin (128). However, due to the extensive innervation of BAT, and the newly presented mechanism for secretin signaling, it is also worthwhile to further explore the afferent neural signaling from BAT to the CNS. In relation to this, it should be noted that the residual BAT in adult humans seems closely connected with the central nervous system by location (Fig. 4). Thus, the potential of peptide exchange between BAT and neighboring neurons warrants further investigation. Moreover, to study whether additional signaling peptides from metabolic tissues such as gut, liver, pancreas, and muscle target BAT with a forwarding effect to the CNS will be an exciting research area in the future. Finally, an important open question is: Which factors are mediating the age-dependent inactivation of BAT? (16, 22). Gradually turning off the energy-consuming activity of BAT might be beneficial from an evolutionary perspective and is thus putatively actively regulated. Identifying novel batokines involved in the above-discussed processes is a promising avenue for the discovery of drug targets promoting metabolic health.

## Additional Information

***Disclosure Summary:*** The authors have nothing to disclose.

## Abbreviations

BAT: brown adipose tissue
BMG: bone morphogenic protein
^18^F-FDG: fluorine-18 fludeoxyglucose
IL: interleukin
MRI: magnetic resonance imaging
NRG4: Neuregulin-4
TGF: transforming growth factor
WAT: white adipose tissue
